# Quantifying Hematopoietic Stem Cell Clonal Diversity by Selecting Informative Amplicon Barcodes

**DOI:** 10.1038/s41598-020-59119-8

**Published:** 2020-02-07

**Authors:** Emily M. Teets, Charles Gregory, Jami Shaffer, James S. Blachly, Bradley W. Blaser

**Affiliations:** 1 The Ohio State University College of Medicine, Department of Medicine, Division of Hematology, The Ohio State University Comprehensive Cancer Center, Ohio, USA; 2 The Ohio State University College of Medicine, Department of Biomedical Informatics, Ohio, USA

**Keywords:** Phylogeny, Haematopoiesis, Haematopoietic stem cells

## Abstract

Hematopoietic stem cells (HSCs) are functionally and genetically diverse and this diversity decreases with age and disease. Numerous systems have been developed to quantify HSC diversity by genetic barcoding, but no framework has been established to empirically validate barcode sequences. Here we have developed an analytical framework, Selection of informative Amplicon Barcodes from Experimental Replicates (SABER), that identifies barcodes that are unique among a large set of experimental replicates. Amplicon barcodes were sequenced from the blood of 56 adult zebrafish divided into training and validation sets. Informative barcodes were identified and samples with a high fraction of informative barcodes were chosen by bootstrapping. There were 4.2 ± 1.8 barcoded HSC clones per sample in the training set and 3.5 ± 2.1 in the validation set (p = 0.3). SABER reproducibly quantifies functional HSCs and can accommodate a wide range of experimental group sizes. Future large-scale studies aiming to understand the mechanisms of HSC clonal evolution will benefit from this new approach to identifying informative amplicon barcodes.

## Introduction

Hematopoietic stem cells (HSCs) are increasingly recognized to be functionally and genetically heterogeneous^1^. HSC clonal diversity has important implications for the study of hematopoiesis and blood disorders^2–6^. Methods for estimating HSC clonal diversity include counting viral integration sites in transduced bone marrow^7^, single-cell or limiting dilution transplantation^8–11^, sequencing transposon insertion sites^12^, SNP analysis in genomic^13,14^ or mtDNA^15^, and genetic barcoding using CRISPR/Cas9 or Cre-Lox-based recombination^16–24^. An ideal method would sample a large fraction of the HSCs in an organism, be able to discriminate many clones with little or no ambiguity, and would mark HSC clones without causing any alteration in cell function. Further, the mark should be induced prior to any experimental intervention, be inherited by all progeny of the HSC, and be detectable using reproducible and cost-efficient means with a minimum investment of labor and computational time. Optimizing these parameters would allow researchers to perform more powerful experiments to address mechanisms of hematopoiesis and leukemogenesis.

The transgenic zebrafish system, Genome Editing of Synthetic Target Arrays for Lineage Tracing (GESTALT), has emerged as a powerful tool for studying cellular phylogeny^16,25^. GESTALT zebrafish carry a single germline copy of a synthetic array consisting of 10 tandem CRISPR/Cas9 target sites. By microinjecting synthetic guide RNAs (sgRNAs) targeting this array with either Cas9 mRNA or recombinant Cas9 protein into the single-cell zebrafish embryo, double strand breaks are induced within the array and then repaired by non-homologous end joining (NHEJ) or microhomology-mediated end joining (MMEJ). The combinatorial effect of editing the 10 sites of the array induces thousands of unique genetic barcodes^16^. Barcoding ceases when all available sites have been edited or when cell division or degradation has reduced the effective concentration of editing reagents below a critical threshold. For experiments using recombinant Cas9 protein, this is estimated to be between 4–5 hours post-fertilization (hpf)^16^. An inducible genetic system has been developed which extends the editing window beyond this time^25,26^. The genetic barcoding can be used to trace cell phylogeny and in theory could be combined with conditional transgenics, mutants, or other genetic or chemical modifications to understand how these experimental conditions affect clonal diversity of the blood system.

There are a number of limitations to the GESTALT method. The fraction of barcoded cells in the GESTALT zebrafish depends on the integrity and quantity of the reagents used and the efficiency with which the injection solution is delivered to the embryo. The NHEJ and MMEJ repair mechanisms can produce stereotypical repair patterns, reducing the actual diversity of barcodes observed. The published bioinformatic analyses do not provide a means to identify these uninformative variants or a systematic approach to exclude samples with a low fraction of informative barcodes. Unique molecular identifiers (UMIs) have been used for sequencing error, PCR error, and PCR bias correction^27^, however the UMI PCR protocol more than doubles the sample preparation time and reagent cost, and the extent to which UMIs improve accuracy over standard PCR has not been demonstrated in this setting.

Here we have developed a sequencing and informatics pipeline optimized for the quantification of amplicon barcodes from genomic DNA which we entitle Selection of Amplicon Barcodes from Experimental Replicates (SABER). By analyzing a large number of zebrafish blood samples, we were able to define a threshold for discriminating informative GESTALT barcodes from uninformative variants and then optimize this threshold through iteration and modeling. Using bootstrap analysis, we provide a method to rationally exclude samples with a low fraction of informative barcodes. We also show that the results of a standard PCR protocol are nearly indistinguishable from a UMI-based PCR protocol. We believe that this experimental method, conceptual framework and reference dataset will be useful to laboratories studying cellular phylogeny, clonal diversity and clonal evolution using amplicon barcodes.

## Results

### Analytical pipeline, sample generation and sequencing

**Figure 1:**
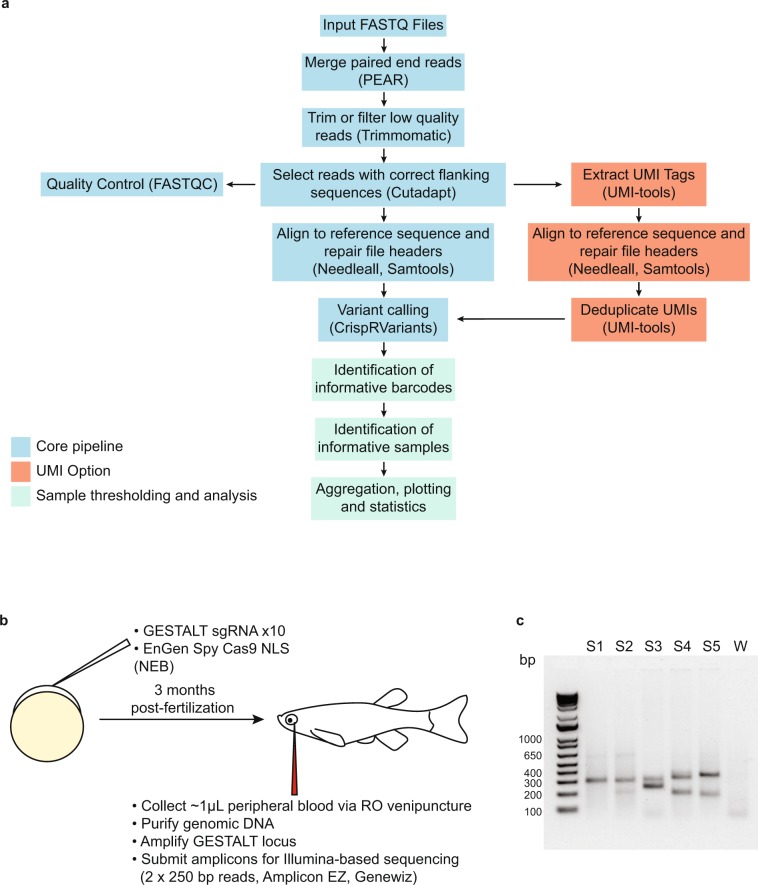
SABER analysis pipeline and experimental outline. (**a**) Major components of the SABER analytical pipeline pictured in blue, red and green boxes. (**b**) Experimental outline for GESTALT barcoding. (**c**) Representative agarose gel electrophoresis of PCR products generated from peripheral blood genomic DNA in GESTALT barcoding experiments. S1-5: 5 samples from the training cohort. W: water control.

SABER is divided into three components: (1) core functions for processing sequence data, aligning to reference sequence and calling variants, (2) an optional module to handle UMI-based PCR amplicons and (3) functions to identify informative barcodes, select samples and perform statistical analysis (Fig. 1a, Supplementary Fig. S1).

To generate samples for this analysis, hemizygous GESTALT transgenic embryos were injected at the single cell stage with an injection mix containing pooled GESTALT sgRNAs and EnGen Spy Cas9 NLS enzyme (NEB) (Fig. 1b). Injected zebrafish were grown to 3 months post-fertilization (mpf) and peripheral blood was collected via retro-orbital (RO) venipuncture. GESTALT barcodes were amplified from genomic DNA via standard or UMI-based PCR. A representative gel with 5 such samples is shown in Fig. 1c. Unfragmented amplicons were sequenced using Illumina-based technology (2 × 250 bp reads, Amplicon EZ, Genewiz) and data was delivered as separate FASTQ files for reads 1 and 2. In the entire dataset, an average of 64,022 ± 13,499 (mean ± S.D.) paired-end reads were obtained per sample and 51,502 ± 14,587 were aligned to the reference sequence after filtering. Supplementary Fig. S2 summarizes the sequencing metrics for the entire dataset.

For this analysis, 56 peripheral blood samples from 3 independent experiments were evenly divided into training and validation sets of 28 samples each. The founder of this line was previously shown to harbor a single insertion of the GESTALT transgene^16^. Each clutch of fish was derived from an outcross of a single GESTALT homozygote and a *casper* zebrafish.

### Standard PCR and UMI-based PCR produce similar GESTALT variant allele frequencies

**Figure 2:**
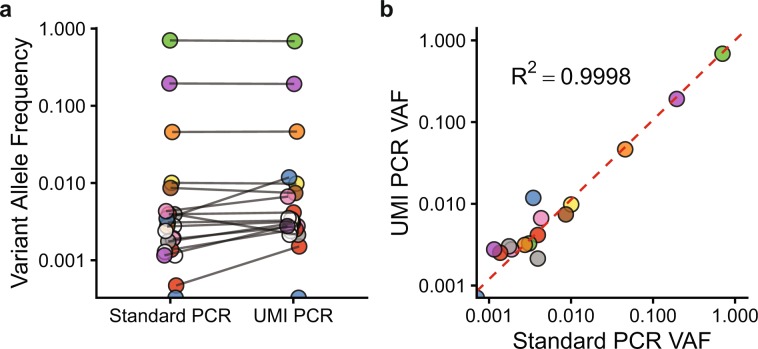
Comparison of Standard and UMI-based PCR protocols. (**a**,**b**) A representative GESTALT blood sample was amplified with UMI-tagged primers using either a standard or UMI-based PCR protocol. (**a**) Variant allele frequencies for the top 20 most common variants in each sample plus the unaltered GESTALT allele. Identical variants are joined; variants missing in either sample are colored white. (**b**) Scatter plot of variant allele frequencies from the same sample. Linear regression model plotted in red (slope = 0.97, intercept = 0.001, R^2^ = 0.9998).

UMI-based PCR techniques reduce sequencing error by generating consensus alleles from reads with identical UMIs and mitigate PCR bias by reducing read groups with the same UMI to a single deduplicated read^27^. This protocol incorporates a series of annealing/extension steps to tag single genomic DNA template molecules with the UMI barcode. Genomic DNA from a subset of 5 samples was processed using the UMI-based PCR protocol. In parallel, a standard PCR (i.e. without the preceding annealing/extension steps), was performed on the same samples using the same UMI-tagged forward primer and reverse primer. All 10 samples (5 UMI PCR and 5 standard PCR) were processed using the core pipeline with UMI deduplication in the UMI PCR samples. Variant allele frequencies for the uncut GESTALT sequence plus the top 20 variants are shown for a representative sample (Fig. 2a). Linear regression analysis showed a high degree of correlation between VAFs derived from standard and UMI-based PCR techniques (average adjusted R^2^: 0.998, N = 5 samples, representative sample with R^2^ = 0.9998 shown in Fig. 2b). In the context of this system, we conclude that UMI-based PCR techniques do not significantly improve the accuracy of variant quantification and so have used the standard PCR protocol and analysis for the subsequent analysis.

### Identification of informative barcodes from GESTALT variants

In cellular phylogenetic terms, a GESTALT sequence variant can be considered an informative genetic barcode if it is unique to the clade of cells descended from the ancestral cell in which the barcoding was performed. The theoretical number of unique barcodes in the GESTALT system is much larger than the number of cells in the embryo at 4–5 hpf, when barcoding likely is complete^16^. However in practice the diversity of barcodes is limited by stereotypical repair patterns arising from the NHEJ and MMEJ double strand break-repair mechanisms^28^. InDelphi was used to predict GESTALT variants with 1% or greater likelihood of occurrence after Cas9 cutting and repair at each target site in isolation^29^. Between 11 and 22 GESTALT variants were predicted across the 10 target sites (Supplementary Fig. S3). However, some variants were predicted to be strongly favored over others, with the most likely predicted variant at each locus having a frequency of between 15.4% and 40.0%. The Shannon entropy calculation for the effective number of barcodes at each locus ranged from 2.5 to 3.3, corresponding to 72,304 potential GESTALT barcodes^30^. This is far lower than the 5.0 × 10^12^ barcodes predicted had each individual variant been equally likely. Barcode diversity is limited further by GESTALT sequences that are never edited and large deletions removing one or more adjacent targets. For two cells from the same organism that carry the same GESTALT sequence, it is impossible to know if that sequence was generated in their last common ancestor cell (meaning that the cells were correctly assigned to the same clade) or if the GESTALT sequence was generated independently in separate clades. The latter GESTALT sequences would be expected to appear repeatedly in a set of samples that can have no cellular phylogenetic relationship (i.e. from different animals), so we developed a method to search for variants shared between samples within the training set and classify them as uninformative GESTALT sequences.

**Figure 3:**
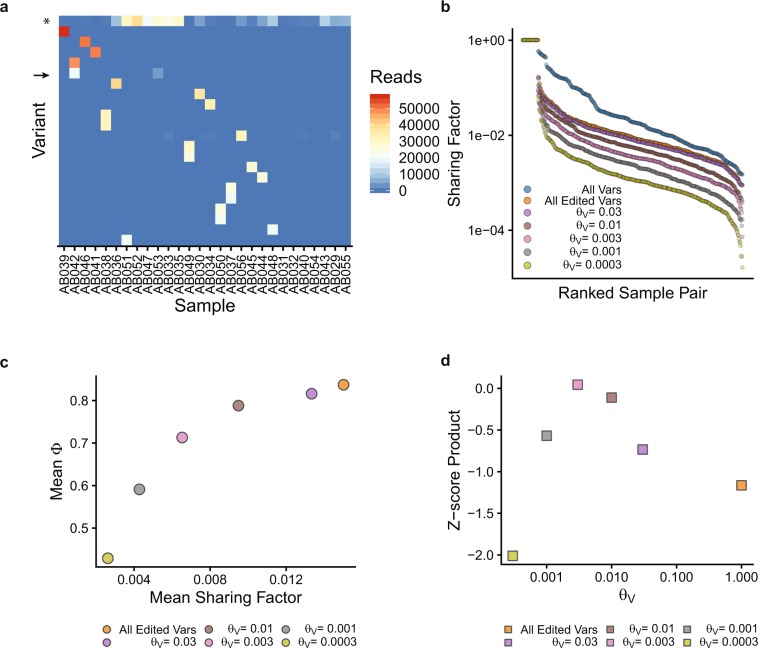
Identification of informative GESTALT barcodes. (**a**) Plot of GESTALT variants by sample. Only variants with read count > 20,000 in any sample are shown. Color indicates read count for each variant. Asterisk indicates the unedited GESTALT allele. Arrow indicates a high-frequency shared allele. (**b**) Sharing Factor curves for all pairwise combinations of samples, grouped by threshold, θ_V_. All Vars: All detected GESTALT variants; All Edited Vars: All edited GESTALT variants with θ_V_ = 1.0. (**c**) The mean Sharing Factor and mean fraction of informative reads remaining after removing common variants and unedited GESTALT alleles (mean Φ) are plotted. (**d**) The Z-score of mean Φ times the Z-score of 1-mean Sharing factor (Z-score Product) is plotted at each value of θ_V_.

The *m*-by-*n* matrix, A, of 28 samples (*m*) and 26,877 unique GESTALT variants (*n*) is highly sparse, suggesting that there are few shared alleles (Supplementary Fig. S4). Unsupervised hierarchical clustering of A showed a group of samples with a large number of reads in a common variant, in this case the unedited GESTALT sequence (asterisk, Fig. 3a). Other than the unedited sequence, one GESTALT variant was shared at relatively high frequency between two samples (variant 125:1I with 20,225 reads and VAF = 0.29 in sample AB042 and 4073 reads and VAF = 0.08 in sample AB053, arrow, Fig. 3a). The 20 most common GESTALT variants detected in the training set are shown in Supplementary Fig. S5. To quantify the degree to which any two samples in the training set shared GESTALT alleles, we developed the Sharing Factor (see Methods/Data Analysis) and calculated this for each pair of samples in the training set. The Sharing Factor can be expressed as the proportion of reads attributed to variants shared between two samples. This is plotted for all pairwise combinations of samples, including all GESTALT variants (edited plus unedited) or only edited GESTALT variants, in Fig. 3b. The mean Sharing Factor in the training set (excluding identical comparisons) was 0.06 for all GESTALT alleles. After removing unedited alleles, the mean Sharing Factor was 0.015.

We sought to cap the maximum frequency permitted for a variant shared between any two samples and to further reduce the Sharing Factor across the dataset. A variant allele fraction threshold (θ_V_) was introduced into SABER with the following heuristic: if a variant is detected above θ_V_ in more than one sample, it is labeled as a “common variant” and excluded from further analysis (Supplementary Fig. S1). A very stringent (low) value for θ_V_ would be expected to identify and exclude a large number of common variants, to minimize the Sharing Factor, and to put a stringent cap on the VAF permitted for any variant shared between two samples; this would also, however, limit the number of informative reads remaining for each sample. To generate a model for minimizing inter-sample variant sharing and maximizing the fraction of informative reads (Φ, defined as the number of reads assigned to informative barcodes divided by the total number of aligned reads) remaining in each sample, the analysis was iterated with θ_V = _0.0003, 0.001, 0.003, 0.01, 0.03, 0.1, and 0.3. Plotting these in descending rank order generated the family of curves shown in Fig. 3b (the curves for θ_V_ = 0.1 and 0.3 are identical to the curve for all edited variants and are omitted for clarity). Mean Sharing Factor and mean Φ values are plotted for the 28 training samples analyzed under each condition (Fig. 3c). To select an optimal value for θ_V_, a standardized measure was calculated by taking the Altman Z-score of the mean Φ at each value of θ_V_ and multiplying this by the Z-score of 1 minus the mean Sharing Factor at each value of θ_V_ (Fig. 3d). The value of θ_V_ corresponding to the maximum of this Z-score product was 0.003. At this threshold setting, variants accounting for more than 0.3% of reads in more than one sample are identified as common variants and excluded. The mean Sharing Factor at this threshold was 0.0065 and the mean Φ was 0.71. All variants not identified as common variants or unedited GESTALT alleles are considered valid GESTALT barcodes.

### Selection of samples based on the fraction of informative barcodes

**Figure 4:**
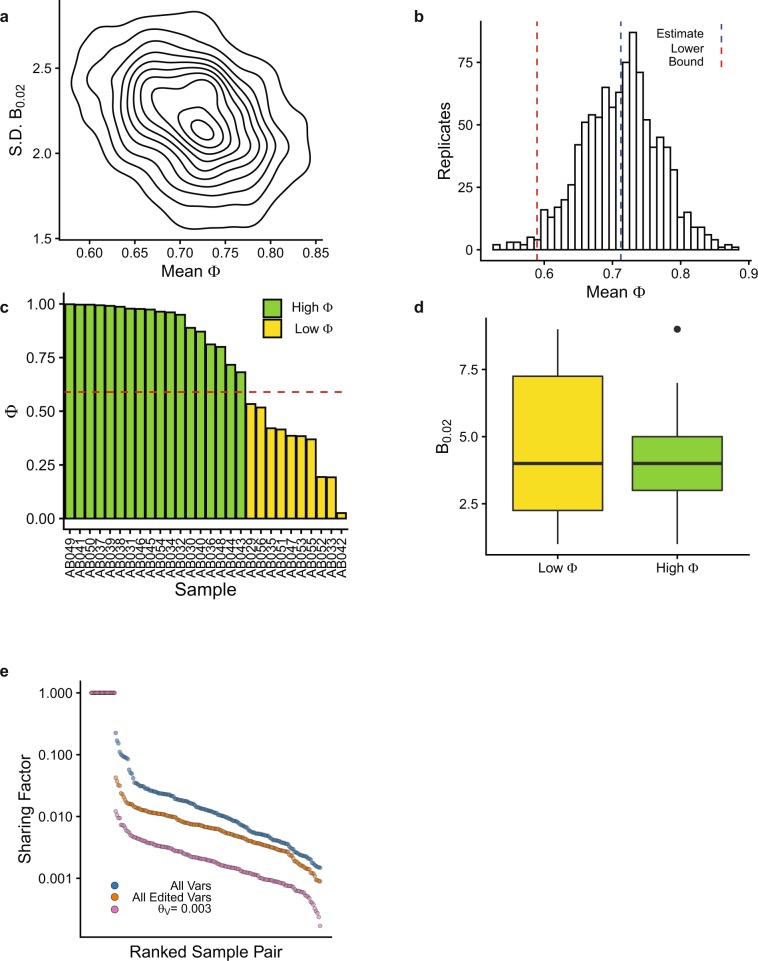
Systematic selection of samples with a high fraction of informative reads. (**a,b**) The fraction of informative reads and number of barcodes with VAF > 0.02 was calculated for each sample in the training set. (**a**) Contour plot of N = 1000 bootstrap replicates for the standard deviation of the number of barcodes with VAF > 0.02 and the mean Φ, r = −0.34. (**b**) Histogram showing the bootstrap estimate and lower bound of the 95% confidence interval using the bias-corrected, accelerated method. (**c**) Fraction of informative reads in each training sample. Red line indicates the confidence interval from b. (**d**) Number of barcodes with VAF > 0.02 in the training samples with low and high fraction of informative reads. Box and whisker plots show median, 1st and 3rd quartiles, and 1.5 x the interquartile range. (**e**) Sharing Factor curves for the samples with a high Φ including all GESTALT variants (blue), all edited GESTALT variants (orange), and all remaining variants at the algorithmically selected final value of θ_V_ (pink).

We next recognized that samples with a low Φ might be less representative of the true number of HSC clones compared to samples with a high Φ. Excluding low Φ samples in a systematic, objective way could improve the accuracy of enumerating HSC clones. For each sample in the training set, Φ and the number of unique valid barcodes with VAF > 0.02 (B_0.02_) was calculated. Bootstrap analysis (N = 1000 repetitions) was performed and the standard deviation of B_0.02_ and mean Φ across all replicates was plotted (*r* = −0.34, Fig. 4a). The inverse relationship between these values supports the notion that samples with a high Φ are less variable and more precise predictors of the number of HSC clones than samples with a low Φ. To objectively identify a cutoff point between high and low Φ samples, the bootstrap estimate and 95% confidence intervals were calculated (mean Φ bootstrap estimate = 0.71, 95% CI = [0.59,0.81], Fig. 4b). The lower bound of the 95% confidence interval, plotted as a horizontal dashed line, classified 18 samples as high Φ and 10 as low Φ (Fig. 4c). The distribution of B_0.02_ for high and low Φ samples was significantly different (Kolmogorov-Smirnov test p = 1.5 × 10^−7^, Fig. 4d). The mean Φ in the low and high Φ samples was 0.34 and 0.92, respectively. The mean Sharing Factor for the 18 high Φ samples was 0.0024, a 25-fold reduction from the original training set (Fig. 4e).

### Enumerating barcoded HSC clones from GESTALT variants

**Figure 5:**
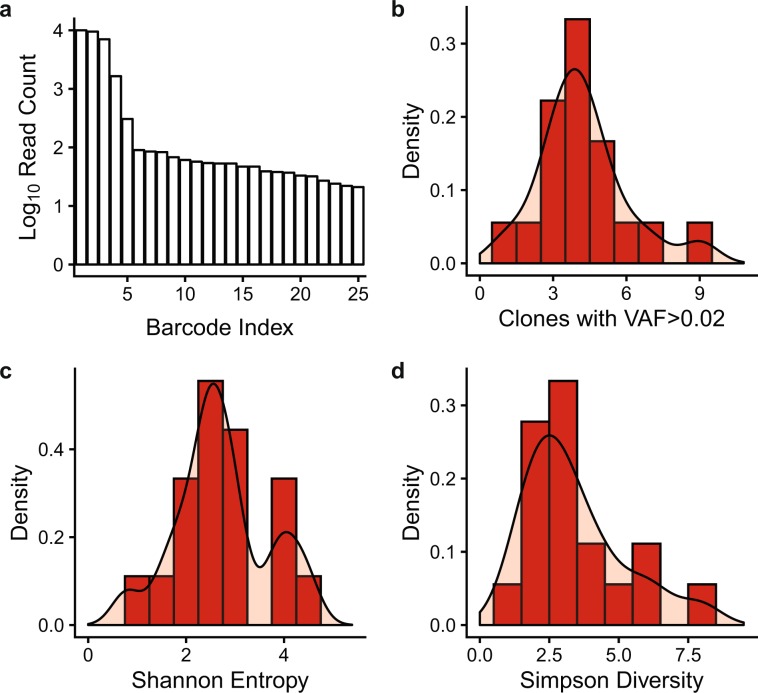
Enumeration of HSC clones from barcoding data. (**a**) Read counts for the top 25 most abundant GESTALT barcodes from a representative sample. (**b**–**d**) Histogram and cumulative density plots for the number of GESTALT HSC clones as determined by (**b**) a flat cutoff of VAF > 0.02, (**c**) Shannon Entropy, and (**d**) the inverse Simpson diversity index.

On average, the number of detected GESTALT barcodes in the training set was large (1254 ± 563 barcodes per sample, range 590–2663). The distribution was heavily right-skewed with 98% of reads assigned to 9 or fewer variants per sample (representative sample with the top 25 of 1046 variants shown, Fig. 5a). This presents a challenge when seeking to quantify HSC clones from variant data. Applying a flat cutoff to variant data (e.g., VAF > 0.02 defines an HSC clone) is currently the standard for quantifying HSC clones in the clinical and research setting^3,4,31^. Weighted averages derived from ecology and population science such as Shannon entropy^30,32^ and the inverse Simpson diversity index^33^ can account for all variants in the sample. However, the weight given to common versus uncommon variants in these schemes is arbitrary. We reasoned that the number of HSC clones detectable in a population of animals should follow a normal distribution and asked whether these calculations (counting clones with VAF > 0.02, Shannon entropy, or Simpson diversity) provided normally distributed data in the training set of samples. For all three calculations, the distribution was not significantly different from normal (Shapiro-Wilk test: p = 0.15, p = 0.79 and p = 0.068 for VAF > 0.02, Shannon entropy and Simpson diversity, respectively, Fig. 5b–d). The Shannon entropy and Simpson diversity calculations were no more resistant to outliers than using the flat cutoff and the distribution for the Simpson diversity index was particularly right-skewed. These data support using the VAF > 0.02 cutoff or the Shannon entropy calculation in this experimental context.

### Analysis of the validation set

**Figure 6:**
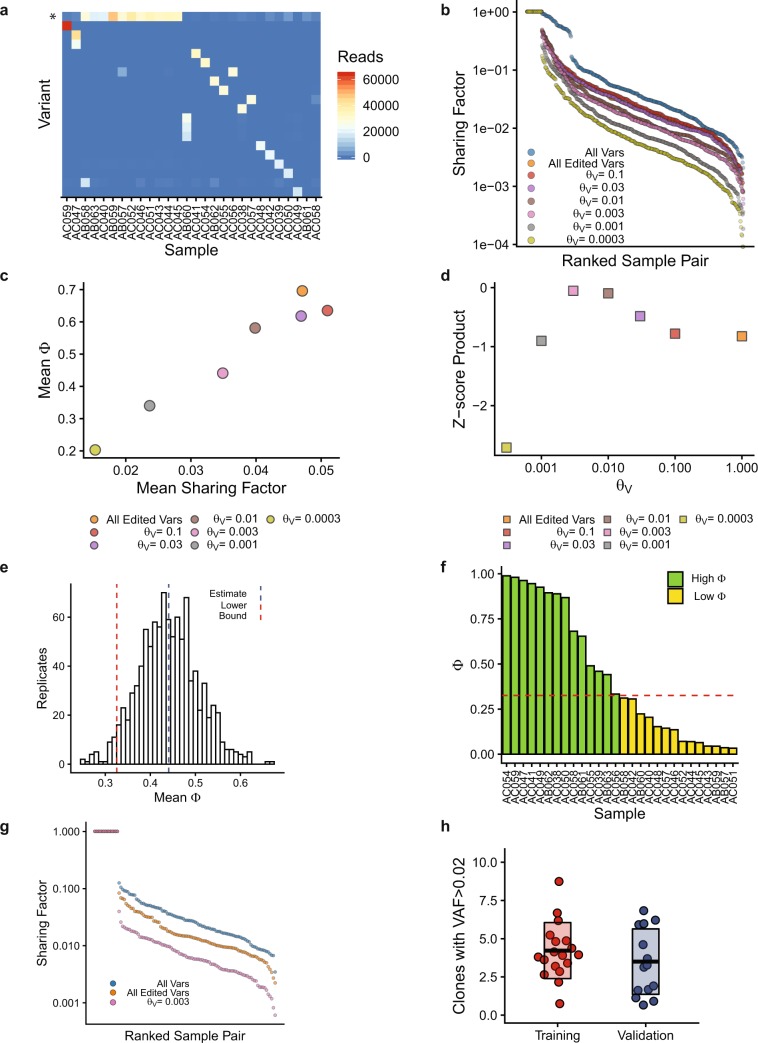
Comparison of training and validation sets. (**a**) Heatmap of GESTALT variant read counts. Asterisk indicates the unedited GESTALT allele. (**b**) Sharing factor curves for all GESTALT variants, all edited GESTALT variants and at indicated values of θ_V_. (**c**) Mean Φ plotted versus mean Sharing Factor at indicated values of θ_V_. (**d**) Z-score product plotted at indicated values of θ_V_. (**e**) Bootstrap analysis of the validation set with estimate and lower bound of the 95% confidence interval. (**f**) Histogram showing samples with a high and low fraction of informative reads. (**g**) Sharing Factor curves at the algorithmically selected final value of θ_V_. (**h**) Number of GESTALT HSC clones with VAF > 0.02 in the training and validation sets. Boxes show mean and standard deviation.

Sequence data from the 28 validation samples were analyzed without UMI deduplication. Between 25,111 and 78,609 reads were obtained per sample (Fig. 6a, Supplementary Fig. S6). Iterative analysis and algorithmic selection of θ_V_ identified an optimal value of 0.003 corresponding to a mean Sharing Factor of 0.035 and a mean Φ of 0.44 (Fig. 6b–d). Bootstrapping identified 14 high Φ samples (mean Φ = 0.75, Fig. 6e) with a mean Sharing Factor of 0.008 (Fig. 6f,g). The number of HSC clones with VAF > 0.02 in the training and validation sets was similar (4.2 ± 1.8 vs 3.5 ± 2.1, respectively, p = 0.31, Fig. 6h).

Two additional independent datasets were analyzed to demonstrate the utility of the SABER experimental and computational framework in identification of inadequately barcoded samples and uninterpretable experiments in general (Supplementary Figs. S7–10). Supplementary Figs. 7 and 8 show data from samples that were generated in a fashion similar to the training and validation sets. Barcoding efficiency was poor in this experiment (95–98% of alleles were unedited, Supplementary Figs. S7a and 8). The algorithmically-selected value for θ_V_ in this experiment was 0.001 (Supplementary Fig. S7b–d). After selecting the most informative samples, the mean Sharing Factor was 0.51 with a mean Φ of 0.01 (Supplementary Fig. S7e). With over half of barcodes shared between any two samples despite removing 99% of the most redundant sequences, SABER clearly identifies this as an uninterpretable experiment. Both conditions elicit warning messages from SABER.

A second dataset was generated using an inducible rather than microinjected CRISPR/Cas9 system with heat-shock induction of barcoding at 26 hpf (Supplementary Figs. S9 and S10)^25^. In this experiment, the labeling efficiency was high, but one editing pattern involving 5 GESTALT sites was identified at very high frequency in 14/16 samples (Supplementary Fig. S9a, arrow indicates common variant, asterisk indicates unedited GESTALT allele; Supplementary Fig S10, variant 122:108D). The algorithmically selected value of θ_V_ was 0.3 (Supplementary Fig. S9b–d). Selection of such a high value by SABER suggests that the majority of barcode sharing is driven by one or a few common alleles, a condition that elicits a warning. After selection of informative samples, the mean Sharing Factor was 0.012 and the mean Φ was 0.55, both of which elicit warnings. Because of the suboptimal labeling, SABER identifies this as an uninterpretable dataset. These parameters (θ_V,_ Φ and mean Sharing Factor) should be reported as quality control measures in any experiment analyzed using SABER.

### Longitudinal analysis of clonal dynamics

**Figure 7:**
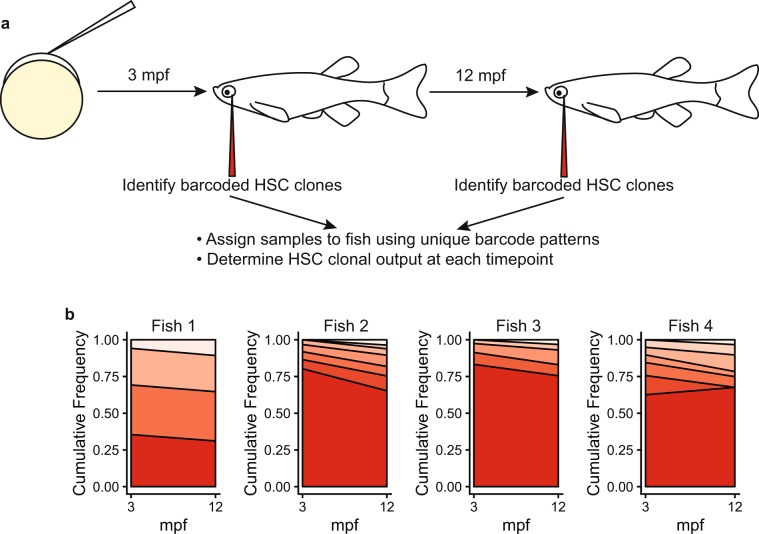
Longitudinal analysis of clonal dynamics. (**a**) Experimental design. (**b**) Stacked area plots showing HSC clones with VAF > 0.02 at 3 and 12 mpf. Each polygon represents a unique HSC clone. The height of each polygon at the 3 and 12 mpf marks corresponds to the frequency of that clone at that time point normalized to a cumulative frequency of 1.0.

To demonstrate the ability to reproducibly detect HSC clones and track the dynamic changes in clonal output over time, a subset of animals in the training cohort were bled again at 12 mpf. GESTALT barcodes were amplified, sequenced and processed using SABER. 3-mpf and 12-mpf samples were matched to unique fish identifiers by manual inspection of the top 5 most frequent barcodes in each sample (Fig. 7a). Barcodes were filtered at each time point to include only those present at a VAF > 0.02 and these were normalized to a cumulative frequency of 1.0. HSC clonal dynamics from 3 to 12 mpf are shown for these 4 unique fish in Fig. 7b.

## Discussion

HSC heterogeneity has been quantified in many terms using *in vitro* assays, single-cell RNA sequencing, transplantation studies and barcoding under conditions of native hematopoiesis^1^. Although conceptually more abstract than transplantation-based assays, studying barcoded native hematopoiesis has the advantage of minimizing potential artifact and bias induced by the requisite stress imposed on the HSC compartment by transplantation. The challenges encountered by various barcoding techniques lie in barcode induction, barcode validity and barcode interpretation. Here we have used the transgenic zebrafish line, GESTALT, and developed SABER as an analytical framework to attempt to overcome these challenges.

Methods to induce barcode labeling in experimental models include CRISPR/Cas9 editing of non-critical regions of DNA^16,17,23–25,34^, random Cre-Lox recombination^20,22,35–37^, random oligonucleotide sequences embedded in transgenes^38^, and transposon mobilization^12^. The percent of hematopoietic cells barcoded using these methods ranges from 30 to over 90. Here we provide evidence that animals with a low fraction of informative barcodes have a higher variance between samples compared to animals with a high fraction of informative barcodes. Using the Sharing Factor statistic and bootstrap analysis, we have developed a systematic, objective method for identifying these less-accurate samples. After removing these from the analysis, the remaining embryos in the training and validation cohorts displayed successful editing of between 75% and 92% of the detected GESTALT sequences.

Barcode validity is determined by the dynamic range of the barcoding system and the resolution of the method employed to “read” the barcodes. Barcodes with a genome-wide distribution such as random transgene insertions^38^, transposon integration sites^12^, CRISPR or Cre-Lox recombination targets present as high-multicopy transgenes^17,20^, or naturally arising SNPs^13^ have a very high dynamic range. The resolution of genetic barcodes is limited only by sequencing error (on the order of 10^−6^ for indels using Illumina-based platforms^39^) whereas the resolution of optical barcodes is limited by the instrumentation and clustering algorithm used. Our data show that the 10 tandem GESTALT CRISPR targets are subject to stereotypical repair patterns which limits the dynamic range of this system. Here we have taken an empiric approach to identifying uninformative GESTALT variants by creating a threshold to define such variants shared across samples and remove them from the analysis. Modeling the fraction of informative reads remaining and the degree of variant sharing by calculating the Sharing Factor at multiple threshold settings allowed us to algorithmically select optimal parameters for the analysis.

The design of genetic barcodes largely dictates the sequencing approach required to read and interpret them. Single-locus barcodes over 300–400 bp total are not compatible with Illumina-based methods and require long-read sequencing technology. Distributed multicopy barcodes require single cell sequencing techniques, which at present severely limit the number of samples that can be assayed^17,23,38^. Because of the single PCR-amplicon structure of the barcode, GESTALT is ideally suited for high-throughput analysis of clonal distribution in many samples. This in turn permits large, well-controlled experiments addressing chemical, genetic or other factors that may affect the clonal distribution but with small effect sizes. Based on our sequencing metrics, throughput and cost per sample could be further improved with 2x or 4x sample multiplexing.

Complex transgenic barcodes like GESTALT have been introduced with the aim of recording the lineage history of each cell in a complex, vertebrate organism^23–25,40^. This requires the genetic recorder to operate continuously over the developmental period of interest and for the recording media to be large and complex enough to encode this history. The molecular and computational requirements for such a system are demanding^41^. Further, if one were to introduce an experimental variable, it may be challenging to disentangle effects of that variable on the biologic system versus effects on the recorder itself. Instead, we have used a pulse-labeling approach in which thousands of barcodes are introduced within the first 4–5 hours after fertilization, prior to gastrulation and well before HSC specification. Any experimental interventions taking effect thereafter (e.g. conditional or tissue-specific transgenes) will not alter barcoding per se and can be interpreted in terms of the effect on the cell lineage being tested. The barcodes detected in the blood samples in this study can be traced to ancestral cells from 4–5 hpf that gave rise to GESTALT-barcoded HSCs. The number of GESTALT-barcoded HSCs detected using this labeling strategy is necessarily lower than strategies where barcoding occurs later in development and the number of cells available for barcoding is larger^20,25^. However, our results are similar to those seen in the original report of the GESTALT line^16^, and extend these findings to a large number of samples with confirmation in an independent cohort of animals.

In this study, we have provided a conceptual framework and analytical approach for quantifying functional GESTALT-barcoded HSC clones from whole blood. It is important to consider that zebrafish red blood cells are nucleated and so this system is insensitive to the hematopoietic lineage bias (e.g. myeloid versus lymphoid) that might occur with the introduction of oncogenes. Sorting cell populations prior to barcode analysis or performing barcode analysis using a single cell sequencing platform^25,26,42–44^ would allow simultaneous recovery of hematopoietic and clonal lineage data. The SABER approach to identifying valid barcodes and selecting samples could also be applied to these methodologies. Indeed, SABER could readily be used to study the clonal diversity of other organ systems or tumor tissues with any single-amplicon transgenic barcode sequence by isolating the cell type of interest through bulk sorting, selection or single cell isolation techniques followed by sequencing of the amplicon barcode. Alternatively, the barcode could be expressed in a tissue-specific manner and the barcode read from cDNA prepared from the organ of interest, analogous to what has been done using the conditional *hsp70l* promoter^25^. As we have shown, SABER can be used to follow longitudinal clonal dynamics and so could be used to study the initiation and progression of hematologic neoplasms or solid tumors and the regeneration of tissues that can be repeatedly sampled such as blood, epidermis and caudal fin structures.

The study of HSC phylogeny and clonal evolution is critically important for our understanding of normal and malignant hematopoiesis^1,2,45,46^. SABER provides a framework to address some of the most fundamental challenges inherent to using amplicon barcodes to study cellular phylogeny: barcode uniqueness, sample selection and experimental quality control. Quantification of non-unique barcodes with the Sharing Factor and rational selection of informative samples are important concepts that are generally applicable to future studies in this field. We anticipate that SABER will be a useful experimental tool to study novel factors that control long-term cellular fate.

## Methods

### Zebrafish

The GESTALT v7 and heat-shock Cas9 (*Tg(hsp70l:zCas9-2A-EGFP,5* × (*U6:sgRNA*))) lines were a kind gift from A. Schier^16^. *Casper* zebrafish were a kind gift from L. Zon^47^. Zebrafish were housed at the Ohio State University Comprehensive Cancer Center. Animals were maintained and experiments were approved and performed under Ohio State University Institutional Laboratory Animal Care and Use Committee (IACUC) protocol 2018A00000012. All work was performed in accordance with American Association for Accreditation of Laboratory Animal Care (AAALAC) guidelines^48^.

### CRISPR/Cas9-based GESTALT barcoding

GESTALT sgRNAs were generated by *in vitro* transcription using established methods^16,49^. A 10 μL injection mix containing approximately 20 ng of each sgRNA (200 ng total), 20 pg control plasmid DNA, 200 ng Tol2 mRNA, 60 pmol EnGen Spy Cas9 NLS recombinant protein (NEB) and phenol red was generated and 1 nL was injected into hemizygous GESTALT embryos at the single cell stage. For experiments using the heat-shock Cas9 line, GESTALT barcoding was performed by incubating embryos at 40 °C for 30 min in embryo medium beginning at 26 hpf.

### Genomic DNA isolation, PCR and amplicon sequencing

Peripheral blood was collected from anesthetized adult (3 mpf) zebrafish by retro-orbital venipuncture and diluted into 50 μL PBS containing 2% FBS and heparin^20^. Genomic DNA was isolated using the Quick-DNA Miniprep Kit (Zymo Research). 5 μL (approximately 250 ng) was used for the standard PCR protocol and 23 μL (approximately 1.15 μg) was used for the UMI-based PCR protocol. Primer sequences are listed in Supplementary Table S1. The standard PCR protocol (using primers v6_7_F_illum and v6_7_R_illum at 0.4 μM each or v6_7_UMI_F and v6_7_R at 0.5 μM each) was initial denaturation at 98 °C × 5 min, 45 cycles of denaturation at 98 °C × 30 sec, annealing at 56 °C × 30 sec and extension at 72 °C × 1 min, with a final extension at 72 °C × 7 min. The UMI-based PCR protocol (starting with v6_7_UMI_F at 4 μM) was denaturation at 98 °C × 5 min followed by 10 cycles of annealing at 56 °C × 20 sec and extension at 72 °C × 1 min. This was followed by 2 rounds of bead purification (Ampure, Beckman-Coulter) to remove excess primer. The entire eluate was then used as a template for a standard PCR reaction with the GC_tag and v6_7_R primers.

GESTALT amplicons were bead purified (Ampure XP, Beckman Coulter) and resuspended at 20 ng/μL. 25 μL (500 ng) samples were submitted for Amplicon-EZ sequencing, an Illumina-based sequencing service compatible with amplicons 150–500 bp in length that does not include a fragmentation step in library preparation (Genewiz). Amplicons were sequenced as 2 × 250 bp paired reads and demultiplexed prior to delivery. Agarose gel electrophoresis images were acquired on an Aplegen gel documentation system with automatic settings and inverted in Photoshop.

### Data analysis

A reproducible analysis pipeline with sample data is freely available at https://github.com/blachlylab/SABER. The sample data are from barcoded GESTALT blood samples similar to those analyzed in Results. SABER is Linux/Mac compatible and requires only installation of Snakemake (via conda) which deploys all other software dependencies. The original R script for the main analysis program, and scripts for comparing clone numbers between experiments and for longitudinal analysis are available at the same site.

Paired-end reads were merged with PEAR, trimmed, and filtered for quality using Trimmomatic (SLIDINGWINDOW:4:15 MINLEN:100). Reads containing incorrect, absent, or multiple flanking primer sequences were filtered out using Cutadapt. Merged reads retained after filtering were aligned to the GESTALT reference sequence using Needleall, an implementation of the Needleman-Wunsch algorithm (gap open penalty = 10, gap extension penalty = 0.25). This aligner is superior to the Burrows-Wheeler aligner for mapping highly divergent sequences such as edited GESTALT variants to a small reference sequence^50^. GESTALT variants were called using the CrispRVariants package using the option split.snv = FALSE to collapse all non-indel mutations into the “no variant” allele^51^.

We sought to quantify the degree to which all pairwise combinations of GESTALT samples shared variants in common. Any variant detected in more than one sample above a certain frequency threshold, θ_V_, might then be excluded from the analysis as non-informative. In this way we could expect to generate a list of unique GESTALT barcodes with a known maximum amount of sharing across the dataset together with the proportion of reads accounting for these unique barcodes. CrispRVariants was used to generate matrices *C* and *P* in which each column refers to a unique sample and each row refers to either the read counts *(C)* or proportion *(P)* for a specific variant in each sample, 1…N, in the dataset (schematic in Supplementary Fig. S1, see Supplementary Figs. S5, S6, S8 and S10 for examples of CrispRVariants output). Rows are identified either as “no variant” or by a systematically generated name derived from the CIGAR string produced by the aligner. Only indel length and position are considered in order to reduce the number of false barcodes arising from base miscalls. SABER then identifies all variants in *P* where the proportional abundance exceeds θ_V_ in more than 1 sample and adds these variant names to a list of common variants. Matrix *C* is split into individual read count tables, *C*_1_*…C*_*N*_ for each sample. Common variants previously identified in *P* are marked on *C*_1_*…C*_*N*_ and their read counts summed for each sample. This sum is added as an additional row labeled “common variant sum” on each table and the individual common variant rows are dropped. The resulting data tables, *C*′_1_*…C*′_*N*_ contain read counts for unedited GESTALT alleles, aggregated common variants and all unique variants as defined by θ_V_.

To optimize the value of θ_V_ through iteration and modeling, we developed a statistic to quantify the magnitude of GESTALT variant sharing across the dataset. For a given variant *v*, sample pair (*p*_1_, *p*_2_*)*, and variant read counts *v*_1_ and *v*_2_, respectively, we define the sharing coefficient for this variant for the pair (*p*_1_, *p*_2_) as:

1 $$s=\frac{2\times \,{\rm{\min }}({v}_{1},{v}_{2})}{{v}_{1}+{v}_{2}}$$

This coefficient has the following properties:

- $0\le s\ll 1$ when $\frac{{v}_{1}}{{v}_{2}}$ or $\frac{{v}_{2}}{{v}_{1}}$ approaches 0.
- $0\ll s\le 1$ when $\frac{{v}_{1}}{{v}_{2}}$ or $\frac{{v}_{2}}{{v}_{1}}$ approaches 1.
- $s=0$ when the variant is unique to one sample in the pair.
- $$s=1$$ when $${v}_{1}={v}_{2}$$.

Considering the matrix,

2 $${\bf{B}}=[\begin{array}{ll}{b}_{11} & {b}_{12}\\ \vdots  & \vdots \\ {b}_{m1} & {b}_{m2}\end{array}]$$

where *m* is the number of distinct GESTALT variants detected in (*p*_1_, *p*_2_) and *b*_*ij*_ is the number of reads of the *i*^*th*^ variant detected in sample *j*, we define the Sharing Factor, *S*, for (*p*_1_, *p*_2_) as

3 $$S=\frac{2\times {\sum }_{i=1}^{m}\,pmi{n}_{i}}{{\sum }_{i=1}^{m}{b}_{i1}+{b}_{i2}}$$

Where:

4 $$pmi{n}_{i}={{\rm{\min }}}_{j=[\begin{array}{cc}1 & 2\end{array}]}{b}_{i,j},\,i=1,\ldots ,m$$

The denominator of Eq. (3) is the sum of all reads in sample pair (*p*_1_*, p*_2_). *S* and *s* are analogous in that *S* = 0 for a pair of samples with no shared variants, *S* = 1 when comparing a pair of identical samples, *S* is close to 0 when shared variants are rare in the pair and *S* is close to 1 when one or more shared variants are common between the pair.

The Sharing Factor, *S*, can be expressed as the proportion of all variants shared between a given pair of samples. SABER calculates *S* and the number of reads assigned to informative barcodes divided by the total number of aligned reads (fraction of informative reads, Φ) for all pairwise combinations of samples in a given dataset with θ_V_ = 0.0003, 0.001,0.003,0.01,0.03,0.1,0.3 and 1. The mean *S* for the dataset (excluding identical comparisons) versus the mean Φ is then plotted for each value of θ_V_ (e.g. Fig. 3c). To select an optimal value for θ_V,_ SABER calculates the Altman Z-score for the mean Φ and 1- mean *S* at each value of θ_V_ and stores these as numeric vectors^52^. The element-wise product of these two vectors is calculated and the maximum of the resulting vector is taken to correspond to the optimal value of θ_V_ (e.g. Fig. 3d).

Bootstrapping was performed using the “boot” package in R by first calculating the number of valid GESTALT barcodes with VAF > 0.02 in each sample and then taking the mean and standard deviation of this value over 1000 replicates. Bootstrap confidence intervals were calculated using the bias-corrected, accelerated method.

Shannon entropy was calculated as

5 $$H^{\prime} =-\mathop{\sum }\limits_{i=1}^{R}\,{p}_{i}\,\mathrm{ln}\,{p}_{i}$$

where R is equal to richness, or the total number of observed barcodes and *p*_*i*_ is equal to the proportion of all barcodes represented by the *i*^*th*^ barcode.

The inverse Simpson Diversity index was calculated as

6 $${}^{2}D=\frac{1}{{\sum }_{i=1}^{R}\,{p}_{i}^{2}}$$

### Statistical methods

Statistical analysis was performed using R, version 3.6.1. All statistical methods are explicitly detailed in Results and in the available software. Box and whisker plots are in the style of Tukey and show median, 1^st^ and 3^rd^ quartiles and 1.5 x the interquartile range. HSC clone data in the training and validation groups were first shown to be normal using the Shapiro-Wilk test and then two-tailed p values were calculated using Student’s t-test. All numeric data are expressed as mean ± standard deviation unless otherwise indicated.

## Supplementary information

Supplementary Information.

## Data Availability

The full datasets generated and analyzed during the current study are available in the Sequence Read Archive (SRA) repository, with the primary accession code PRJNA563355 (http://www.ncbi.nlm.nih.gov/bioproject/563355).
