# Supplementary material for: Quantifying Hematopoietic Stem Cell Clonal Diversity by Selecting Informative Amplicon Barcodes
Source: Sci Rep. 2020 Feb 7;10:2153. doi: 10.1038/s41598-020-59119-8 (PMC7005852; doi:10.1038/s41598-020-59119-8)
Supplement: Supplementary file 1 — Supplementary Information. [file 41598_2020_59119_MOESM1_ESM.pdf]

Supplementary Information for

Quantifying Hematopoietic Stem Cell Clonal Diversity by Selecting Informative Amplicon Barcodes

Emily M. Teets[1†], Charles Gregory[1†], Jami Shaffer[1], James S. Blachly[1,2‡], Bradley W. Blaser[1*‡]

[1]The Ohio State University College of Medicine, Department of Medicine, Division of Hematology; The Ohio State University Comprehensive Cancer Center

[2]The Ohio State University College of Medicine, Department of Biomedical Informatics

Supplementary Figure S1

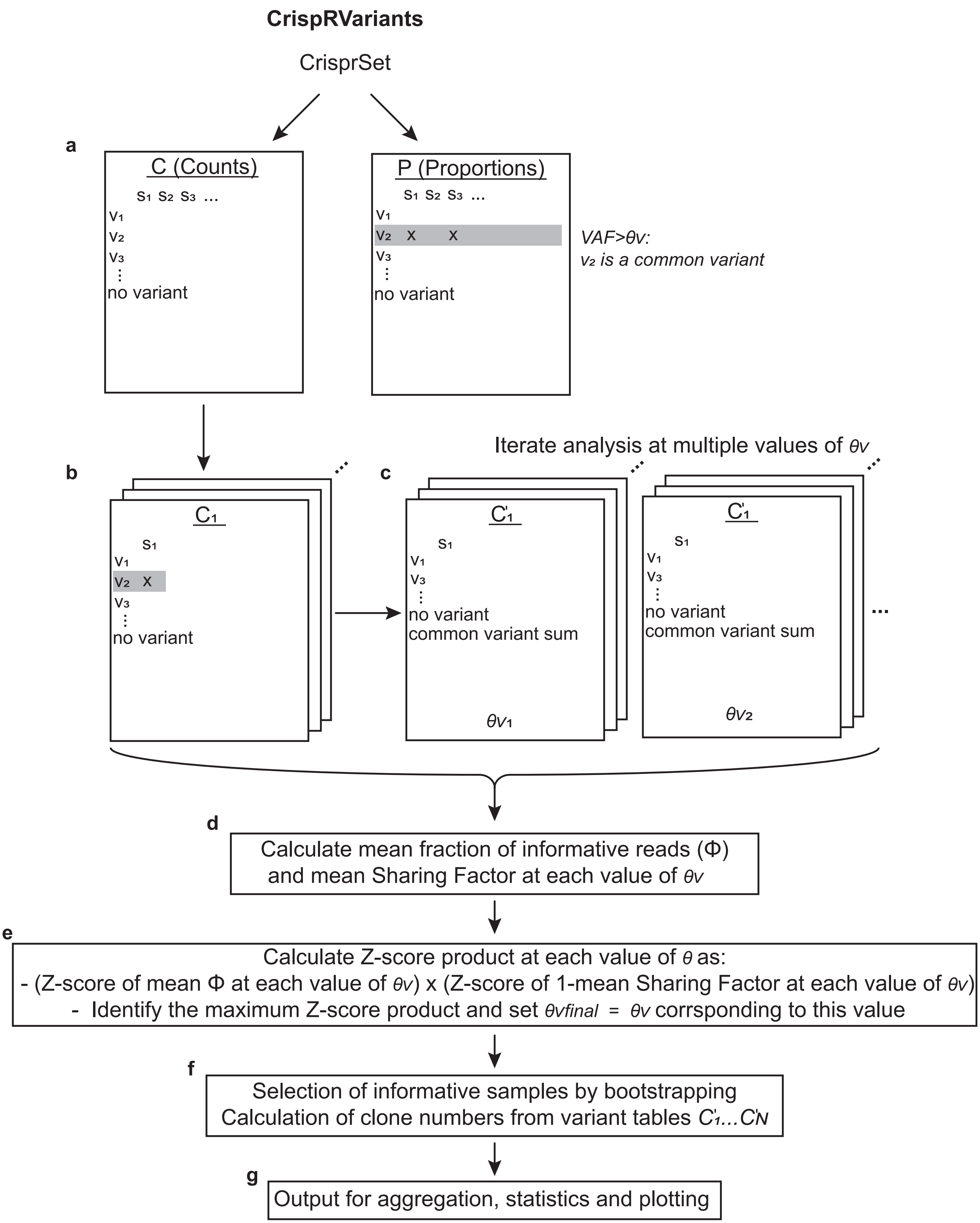

Supplementary Figure S2

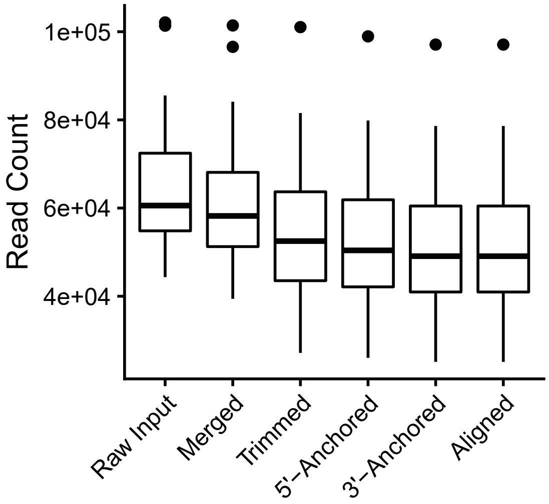

Supplementary Figure S3

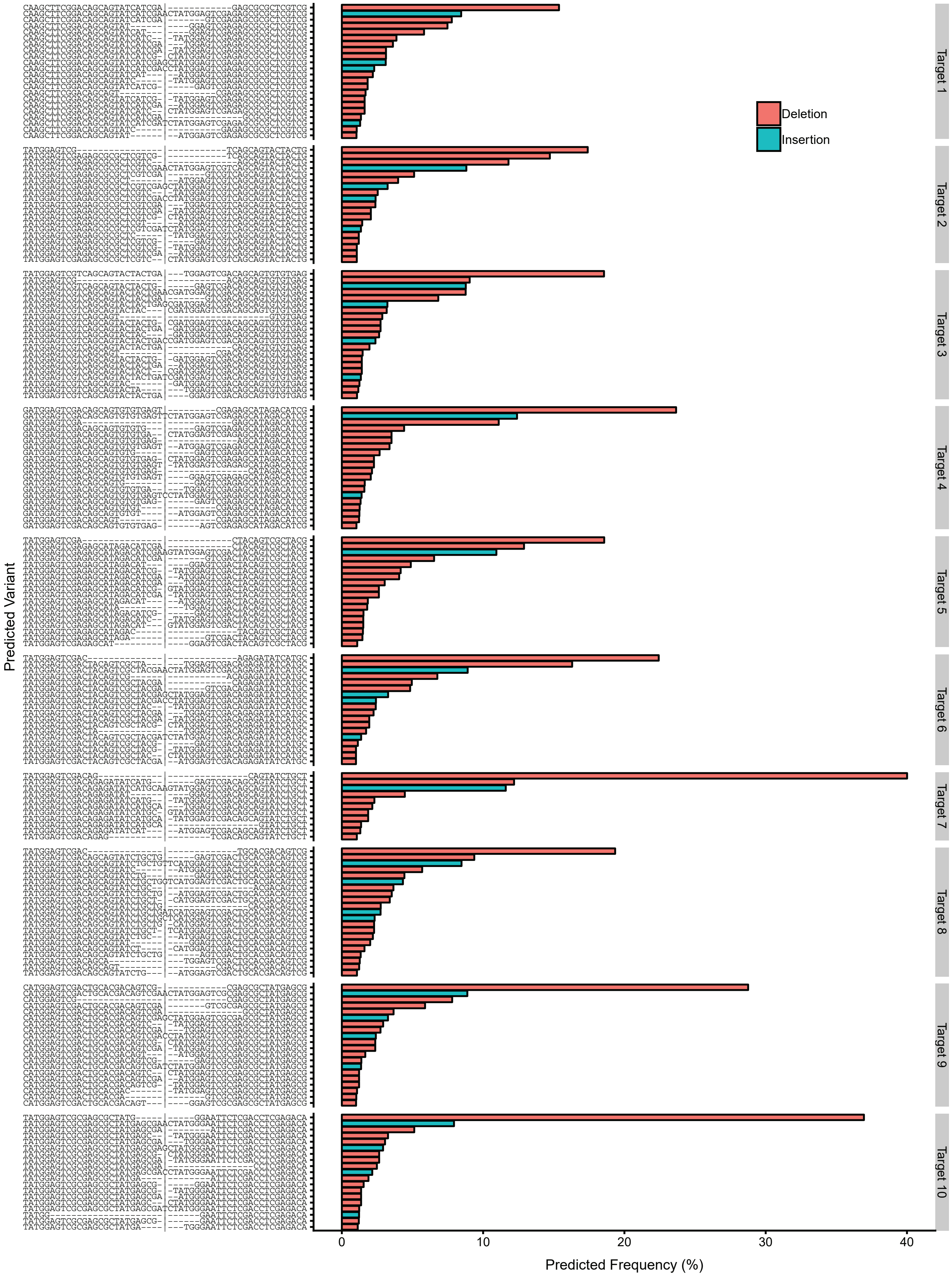

Supplementary Figure S4

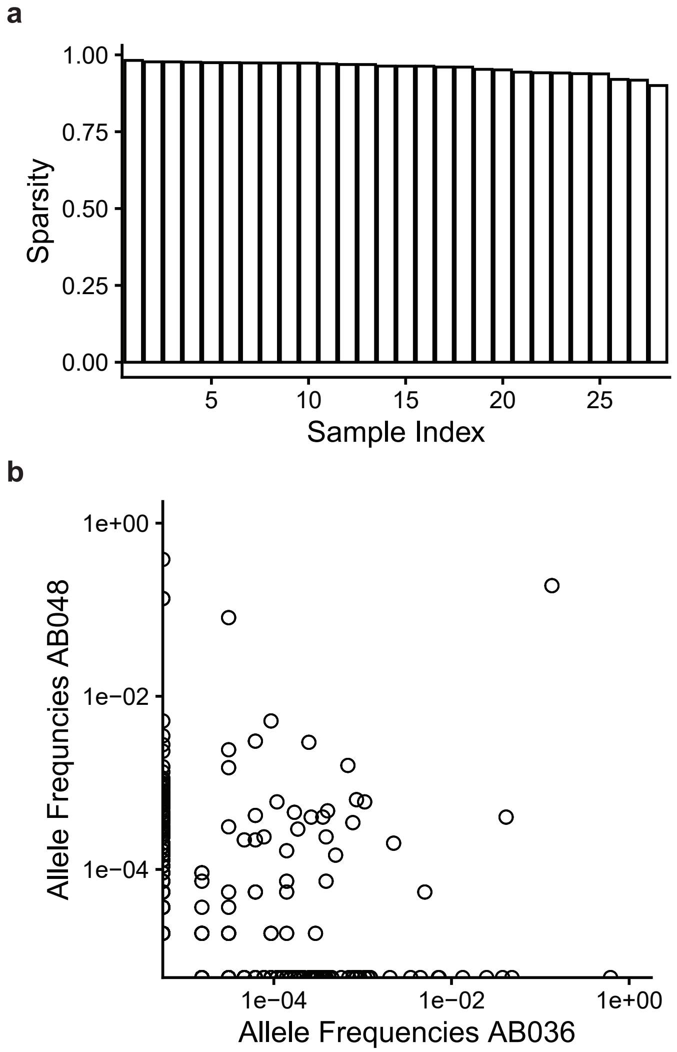

Supplementary Figure S5

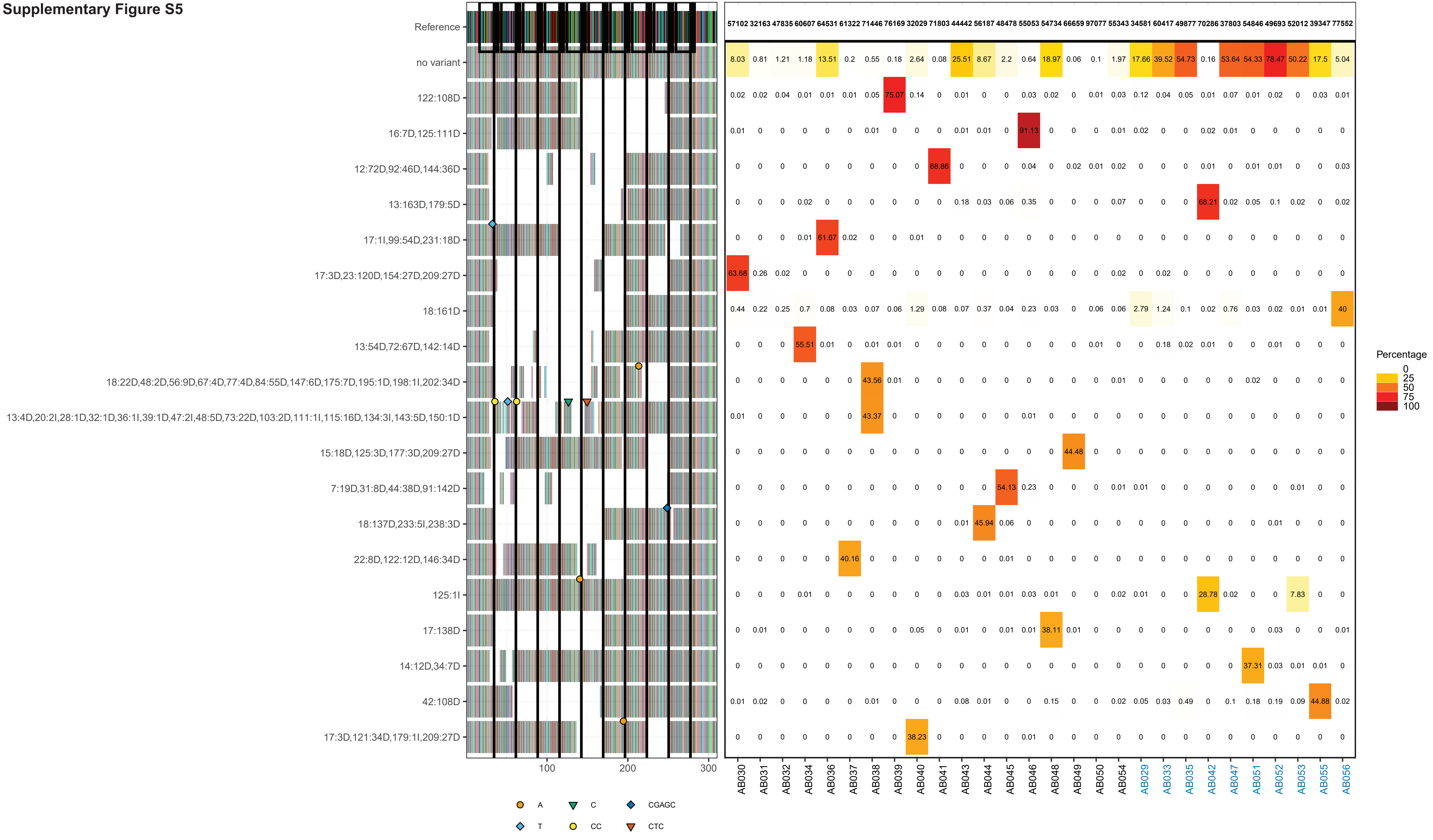

Supplementary Figure S6

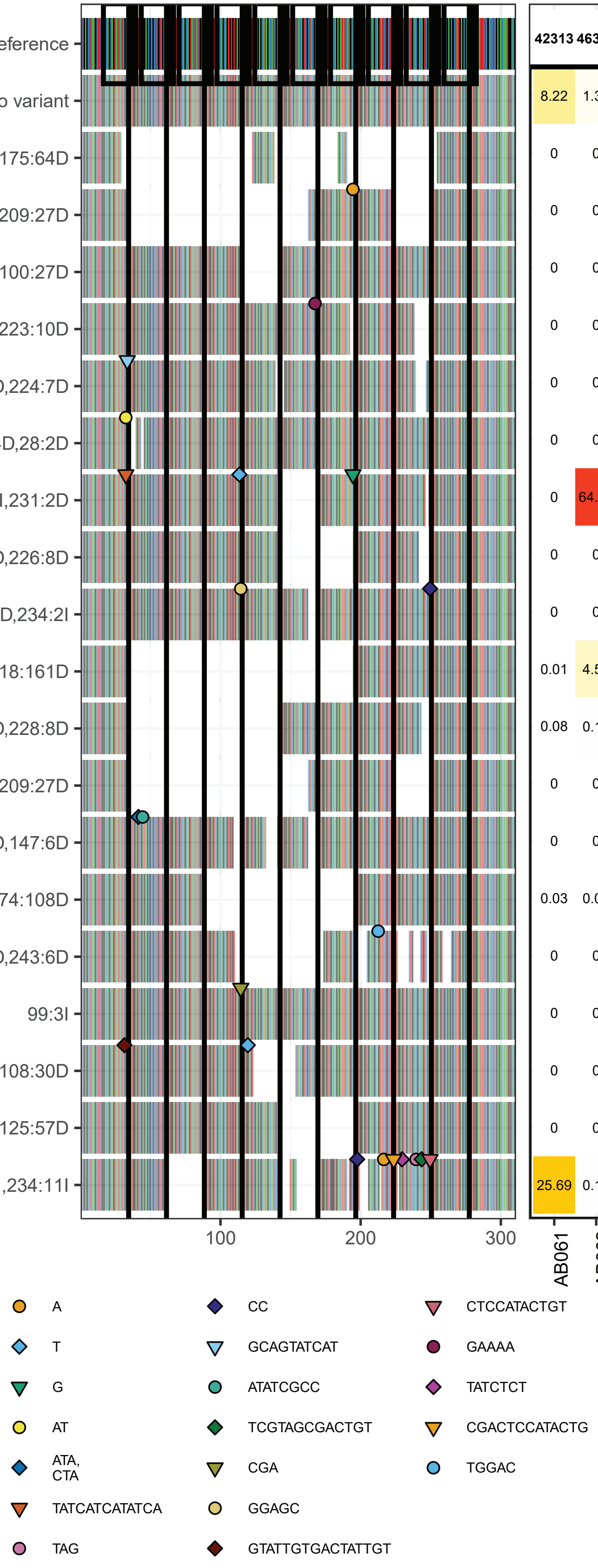

	AB061	AB062	AB063	AC038	AC039	AC041	AC047	AC049	AC050	AC054	AC055	AC056	AC058	AC059	AB057	AB058	AB059	AB060	AC040	AC042	AC043	AC044	AC045	AC046	AC048	AC051	AC052	AC057
	42313	46344	43837	41176	51919	38673	74138	41031	45328	41447	59620	45164	25111	67750	51650	68704	51520	78609	36395	42217	35164	40466	38169	43056	40758	36462	43791	33915
no variant	8.22	1.33	44.66	3.66	2.25	1.29	2.63	0.27	9.99	0.14	0.35	1.25	5.8	1.32	57.25	41.74	92.69	0.13	54.07	6.28	90.97	82.35	86.12	71.38	0.34	94.65	89.25	2.12
14:93D,123:45D,175:64D	0	0	0	0	0	0	0	0	0	0	0	0	0.03	94.66	0	0	0	0	0	0	0.05	0	0	0	0	0.02	0	0
18:129D,179:1I,209:27D	0	0	0	0.01	0.03	0.01	58.46	0	0	0	0.02	0	0	0	0	0	0	0	0	0	0.01	0	0	0.01	0.19	0.01	0.05	0
100:27D	0	0	0	0	0.01	0	0	0.01	0.01	0	0.01	64.18	0.01	0	13.68	0	0	0	0.04	0.01	0	0.92	0	0.01	0	0.01	0.02	0.22
152:5I,177:3D,223:10D	0	0	0	0	0	84.24	0	0	0	0	0	0	0	0	0	0	0	0	0	0	0	0	0	0.02	0	0	0	0.02
18:10I,124:6D,224:7D	0	0	0	0.01	0	0	0	0	0.01	76.8	0.02	0	0.1	0	0	0	0	0	0	0	0	0	0	0.01	0	0	0	0
17:2I,20:4D,28:2D	0	0	0	0	0	0.02	0	0	0	0	0	0	10.58	0	0	0	0	0	0	0	0	0	0	0	0	0	0	80.99
17:13I,98:1I,125:29D,179:1I,231:2D	0	64.05	0.05	0	0	0	0	0	0	0	0	0	0	0	0	0	0	0	0	0	0	0	0	0	0	0	0	0
126:53D,226:8D	0	0	0	0	0.01	0	0	0	0	0.01	49.1	0.09	0.32	0	0	0	0	0	0	0	0	0	0	0	0	0	0	0.13
99:5I,147:6D,234:2I	0	0	0	67.63	0.03	0.01	0	0	0	0.01	0	0	0	0	0	0	0	0	0	0	0	0	0	0.01	0	0	0	0
18:161D	0.01	4.56	1.05	0.9	2.22	1.3	0.13	0.14	0.38	0.3	0.05	0.15	1.89	0.22	0.13	24.8	0.14	0.02	0.56	0.22	1.58	1.93	0.68	2.92	0.04	0.2	0.11	0.31
17:111D,228:8D	0.08	0.12	0	1.1	0.03	0	0.06	0.8	0.25	0	0	0	0	0	0	0	0.01	31.88	0.04	0.03	0.01	0.02	0.04	0.03	0	0	0.03	0
18:129D,209:27D	0	0	0	0	0.01	0	32.42	0	0	0	0.01	0.02	0	0	0	0	0	0	0	0	0.01	0.01	0	0	0.11	0	0.02	0.04
18:1D,26:3I,29:8I,94:8D,117:8D,147:6D	0	0	0	0	0	0	0	0	0	0	0	0	0.41	0.01	0	0	0	0	0	0	0	0	0	0	56.05	0.01	0.03	0.02
74:108D	0.03	0.01	0.29	0.07	37.79	0.21	0.02	0	0.15	0.01	0.07	0.07	0.07	0	0.22	0.39	0.31	0	0.38	0.05	0.32	0.35	0.31	0.21	0.02	0.38	0.38	0.01
17:3D,95:63D,179:10D,197:5I,208:1D,211:8D,222:5D,232:4D,243:6D	0	0	0	0	0	0.01	0.04	0	0	0	0	0	0	0	0	0	0	0	0	50.33	0.28	0.32	0.03	0.01	0	0.01	0	0
99:3I	0	0	0	0.01	0.01	0	0	0	41.05	0	0.01	0.01	0	0	0	0	0	0	0	0.01	0	0.01	0	0	0	0.09	0.01	0.02
16:16I,104:1I,108:30D	0	0	0	0	0	0	0	40.56	0.01	0	0	0	0	0	0	0	0	0	0	0	0	0	0	0	0	0.01	0	0
125:57D	0	0	0	0	0	0	0	0	0.02	0	0	0.01	0.23	0	0	0	0	0	0	0.45	0.01	0.04	0.01	0.01	28.06	0.03	0.03	0
47:27D,126:8D,139:13D,175:1D,182:2I,184:6D,198:1D,201:1I,208:13I,211:1D,214:7I,224:3I,228:13I,234:11I	25.69	0.17	0.03	0.02	0.02	0	0.01	0.02	0	0	0	0	0	0	0	0	0	0	0	0.04	0.04	0.07	0.03	0.44	0	0.02	0	0.04

Percentage: 0, 25, 50, 75, 100

Supplementary Figure S7

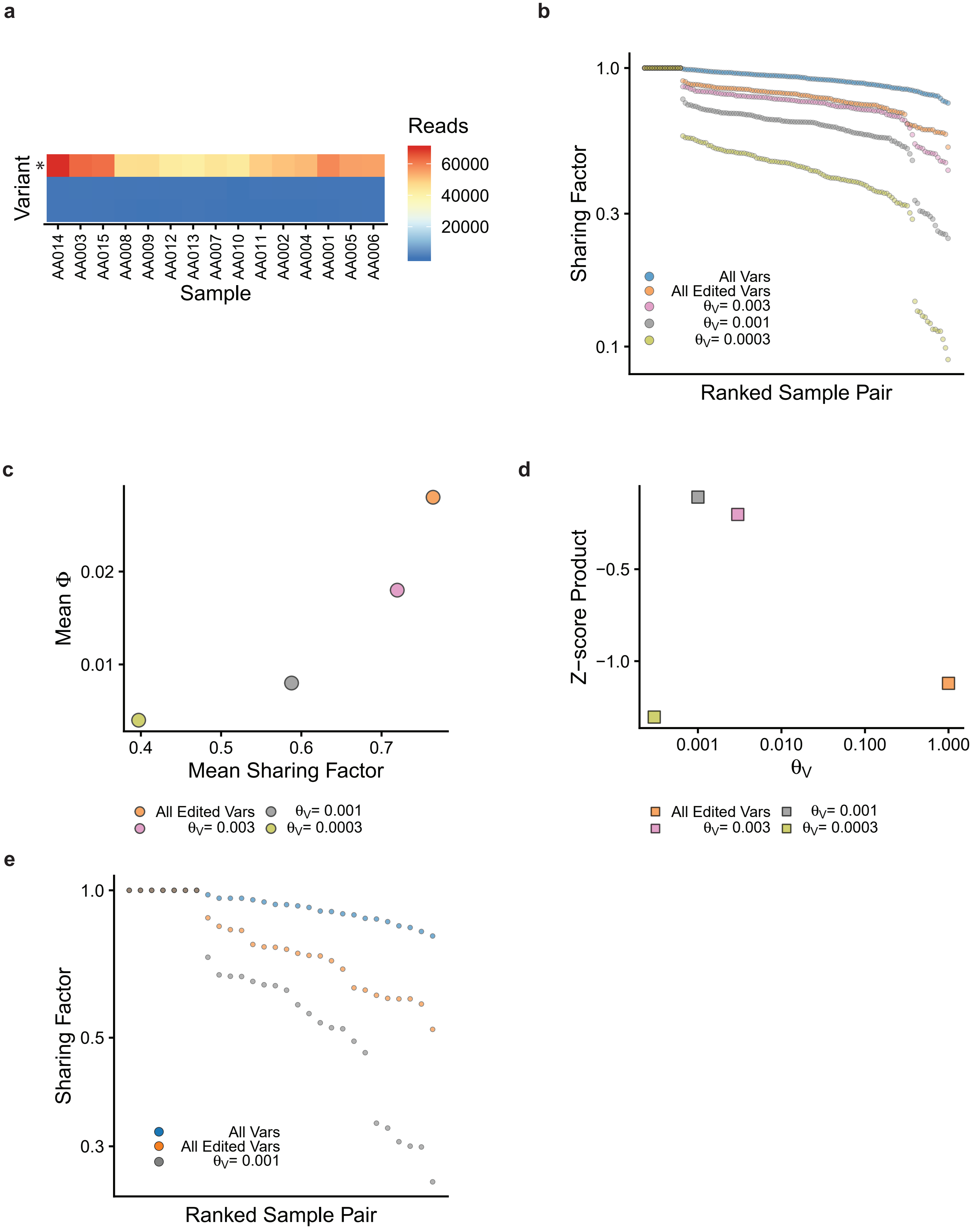

Supplementary Figure S8

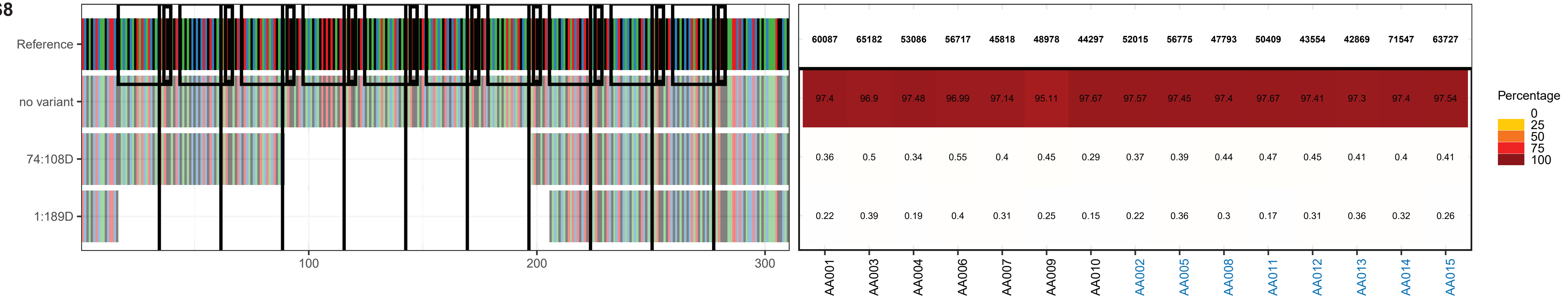

Supplementary Figure S9

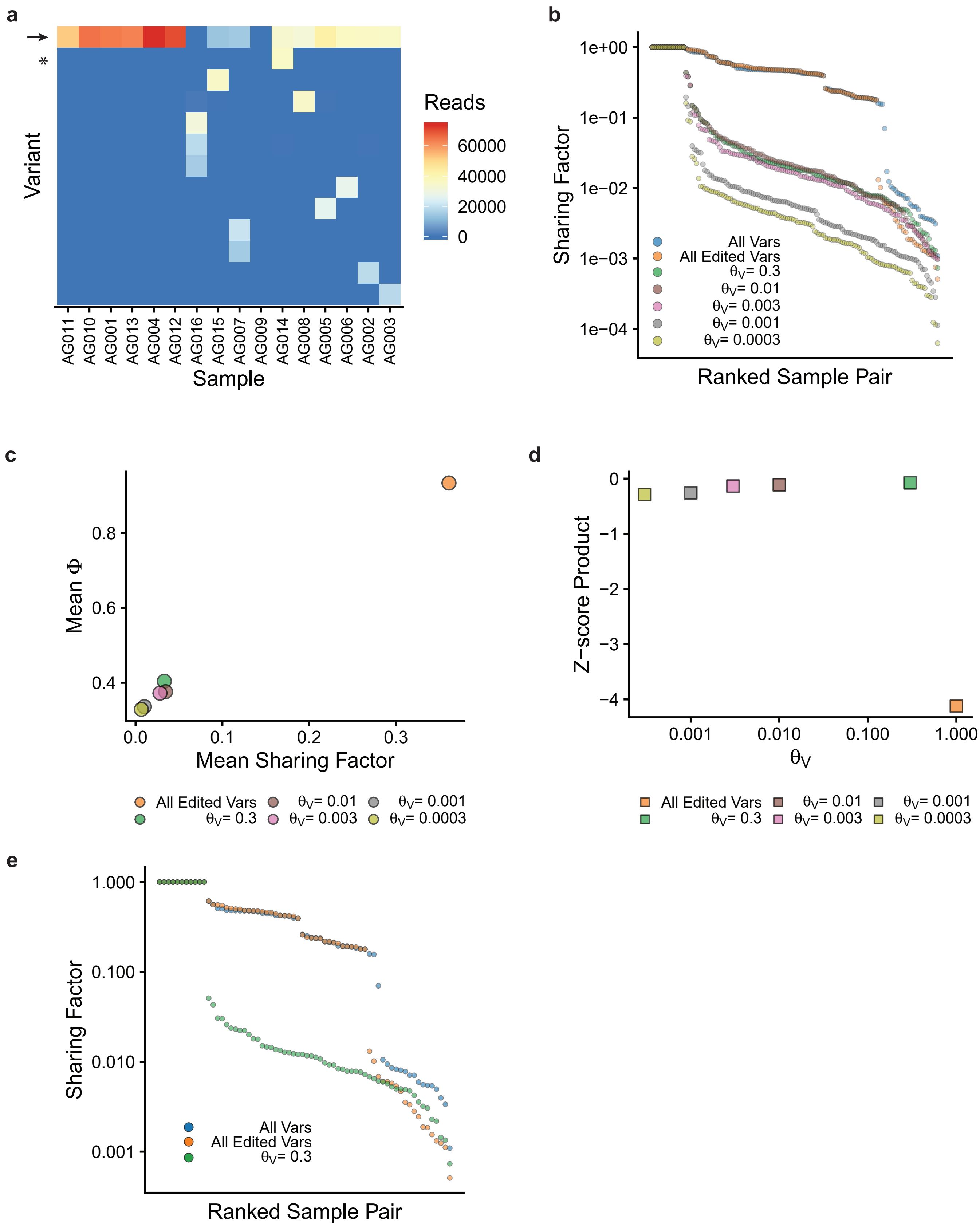

Supplementary Figure S10

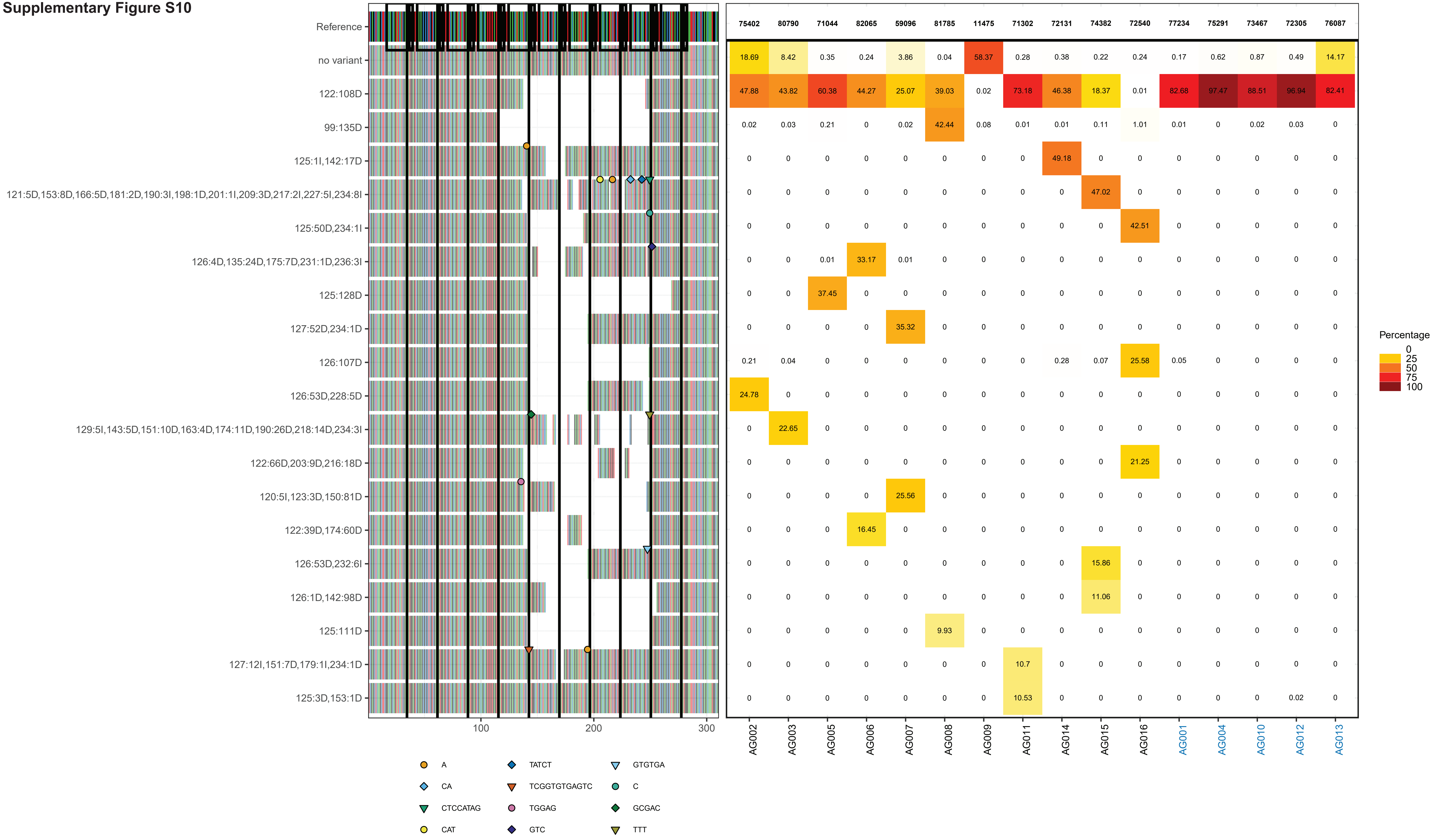

Supplementary Figure Legends

Supplementary Figure S1. Threshold optimization and identification of informative barcodes. a) CrispRVariants produces two data tables, *C* and *P*, containing variant counts and proportions, respectively, for each sample. Variants with an allele frequency in *P* that are greater than θv are marked as common variants. b) Table *C* is split into tables $C_1 \ldots C_N$ to include single samples from the dataset. c) Common variants are identified, removed from the variant list and their counts are summed to form table $C'_1 \ldots C'_N$. This process is iterated at multiple values for θv. d) The mean fraction of informative reads and mean Sharing Factor are calculated for the dataset at each value of θv. e) The Z-score product is calculated as shown in order to select the optimal value of θv. f) Bootstrapping is used to identify samples with a high fraction of informative reads and clone numbers are calculated as described in the main text. g) SABER outputs raw variant counts and calculated HSC clone numbers which can be aggregated and plotted using any software program.

Supplementary Figure S2. Read counts for training and validation datasets analyzed with the core pipeline. Box and whisker plots show median, 1st and 3rd quartiles, and 1.5 x the interquartile range.

Supplementary Figure S3. Predicted GESTALT target editing patterns. InDelphi was used to predict individual GESTALT target repair patterns after CRISPR/Cas9 editing. Left panels show predicted nucleotide sequence; right panels show predicted frequency. Variants with predicted frequency greater than 1% are shown.

Supplementary Figure S4. Shared GESTALT variants are uncommon. a) Matrix sparsity was calculated for each sample in the training set and plotted in descending rank order. b) Scatter plot

of variant allele frequencies for GESTALT variants detected in two representative samples. 55/1821 variants had non-zero allele frequencies in both samples.

Supplementary Figure S5. GESTALT variants and allele frequencies in the training set. Left panel: The top 20 most common GESTALT variants are graphically represented and labeled by variant name. Vertical black lines represent predicted cut site for each target. Right panel: Heatmap of variant allele frequencies. Column headers indicate the number of reads for each sample. Column bases indicate sample names. Black text indicates samples with a high fraction of informative reads and blue text indicates excluded samples with a low fraction of informative reads as determined by SABER.

Supplementary Figure S6. GESTALT variants and allele frequencies in the validation set. Left panel: The top 20 most common GESTALT variants are graphically represented and labeled by variant name. Vertical black lines represent predicted cut site for each target. Right panel: Heatmap of variant allele frequencies. Column headers indicate the number of reads for each sample. Column bases indicate sample names. Black text indicates samples with a high fraction of informative reads and blue text indicates excluded samples with a low fraction of informative reads as determined by SABER.

Supplementary Figure S7. Example of an experiment with poor GESTALT barcoding. a) Heatmap of variant counts for each sample in the dataset. Asterisk indicates the unedited GESTALT allele. b) Sharing Factor curves at indicated values of θv. c) Mean Φ and mean Sharing Factor at the indicated values of θv. d) Z-score product plotted against θv. The optimal value for θv selected by SABER was 0.001. e) At $\theta v = 0.001$, the mean Sharing Factor was 0.51 in the selected samples.

Supplementary Figure S8. GESTALT variants and allele frequencies in an experiment with poor barcoding. The plot layout is similar to Supplementary figures S5 and S6.

Supplementary Figure S9. Example of an experiment with a high-frequency common GESTALT variant. a) Heatmap of variant counts for each sample in the dataset. Asterisk indicates the unedited GESTALT allele. Arrow indicates allele 122:108D. b) Sharing Factor curves at indicated values of θv. c) Mean Φ and mean Sharing Factor at the indicated values of θv. Note that after elimination of allele 122:108D with $\theta v = 0.3$, there is little further reduction in mean Φ or Sharing Factor. d) Z-score product plotted against θv. The optimal value selected by SABER was 0.3. e) At $\theta v = 0.3$, the mean Sharing Factor was 0.012 in the selected samples.

Supplementary Figure S10. GESTALT variants and allele frequencies in an experiment with a high-frequency common GESTALT variant. The plot layout is similar to Supplementary Figures S5, S6 and S8.

Supplementary Table

Supplementary Table S1. Primer sequences used in this study.

Name	Sequence (5'-3')
v6_7_F_illum	ACACTCTTTCCCTACACGACGCTCTTCCGATCTTCGAGCTCAAGCTTCGG
v6_7_R_illum	GACTGGAGTTCAGACGTGTGCTCTTCCGATCTCTGCCATTTGTCTCGAGGTC
v6_7_UMI_F	CGCAGAGAGGCTCCGTGNNNNNNNNNNCTCAGATCTCGAGCTCAAGCTTCGG
v6_7_R	CTGCCATTTGTCTCGAGGTC
GC_tag	CGCAGAGAGGCTCCGTG
